# Length of Interdialytic Intervals Affects Morbidity and Mortality in Chronic Haemodialysis Patients

**DOI:** 10.21767/2472-5056.100038

**Published:** 2017-06-15

**Authors:** Jalal E Hakmei, Paul J Nietert, Wayne R Fitzgibbon, Michael E Ullian

**Affiliations:** Medical University of South Carolina, Ralph H Johnson Veterans Administration Hospital, South Carolina, USA

**Keywords:** Haemodialysis, Interdialytic interval, Cardiovascular outcomes

## Abstract

**Background:**

Chronic in-center hemodialysis (HD) patients may experience more morbidity and mortality after the weekend. Since our Veterans Administration Hospital HD unit is closed on the weekend, non-traditional HD schedules were created. Some schedules contained a 4-day weekend compared to the usual 3-day weekend. We hypothesized that there are more frequent cardiovascular events (CVEs) and higher mortality after longer interdialytic intervals.

**Methods:**

Patients (n=85) were placed on HD schedules as they became available. The usual interdialytic interval group consisted of patients dialyzing on Mon-Wed-Fri or Mon-Tue-Fri (longest interdialytic gap 3 days, n=29), and the long interdialytic interval group consisted of patients dialyzing on Mon-Wed-Thu, Mon-Tue-Thu, Tue-Wed-Fri, or Tue-Thu-Fri (longest interdialytic gap 4 days, n=56).

**Results:**

All-cause mortality was not different between groups, and CVEs occurred more frequently in the usual interdialytic interval group (maybe due to higher mean potassium and phosphorus concentrations). However, within each group, a similar pattern of CVE occurrence as a function of time after dialysis was observed. Compared to CVEs occurring during the 2 days after HD (the lowest frequency), CVEs occurred 2–3 times more frequently during and immediately after HD and 5–7 times more frequently during the third and fourth days after HD. The greatest risk of CVE occurred during the fourth day after HD, which exists only in the long interdialytic interval group.

**Conclusion:**

In chronic HD patients, CVEs are most likely to occur after the longest interdialytic intervals.

## Introduction

Most end-stage renal disease patients maintained on incenter chronic haemodialysis (HD) dialyze on Monday-Wednesday-Friday (Mon-Wed-Fri) or Tuesday-Thursday-Saturday (Tue-Thu-Sat) schedules. A number of studies have suggested that there may be more cardiovascular events (CVEs) and deaths after the weekend (i.e., on Mon and Tue, respectively) than during the rest of the week in adults [1–8] and in children [9]. The interdialytic interval that includes weekends (3 days (d)) is larger than the interdialytic intervals during the workweek (2 d). The increase in morbidity and mortality after the weekend may be related to larger increases in serum potassium concentration with resulting arrhythmias or larger increases in extracellular fluid volume, which might result in myocardial stunning [10] from more aggressive ultrafiltration in attempts to correct the volume overload [11–13]. Also, cardiac arrhythmias and sudden cardiac death were noted to be more common after the long interdialytic interval than after shorter interdialytic intervals [14].

Since our Veterans Administration hospital (VAH) affiliate HD unit is closed on the weekend, non-traditional HD schedules were created to allow for 3 HD sessions during each week for each patient. Some schedules contained the long interdialytic interval of 4 d rather than the traditional long interdialytic interval of 3 d. This natural experiment allowed us to examine the relationship between interdialytic interval and CVEs/mortality in a unique manner. We hypothesized that, compared to shorter interdialytic intervals, longer interdialytic intervals result in more frequent CVEs and higher mortality in chronic incenter HD patients.

## Methods

### Patient selection

We reviewed the charts of all patients undergoing chronic outpatient maintenance HD at the Ralph H Johnson VAH in Charleston South Carolina between 2002, when the electronic medical record (Computerized Patient Record System) was first used for recording data from HD treatments, and October 2015. Those dialyzing on Mon-Wed-Fri and Mon-Tue-Fri constituted the usual interdialytic interval group (U-IDI), with no more than 3 d between HD sessions, and those dialyzing on Mon-Wed-Thu, Mon-Tue-Thu, Tue-Wed-Fri, and Tue-Thu-Fri constituted the long interdialytic interval group (L-IDI), with 4 d at times between HD sessions. No patients dialyzed on a Mon-Thu-Fri schedule. Patients were placed on U-IDI or L-IDI HD schedules as time slots became available or as the patients desired; patients were not assigned to HD schedules for medical reasons. Clinical events and laboratory values were reviewed on patients older than 18 years of age after 3 months on chronic HD and continued until renal transplantation, death, change in dialysis modality, or transfer to another chronic HD unit. Patients whose HD schedule changed were excluded from analysis.

### Outcomes

Outcomes consisted of: 1) all-cause mortality in U-IDI versus L-IDI, 2) the frequency of CVEs in U-IDI versus L-IDI, and 3) the frequency of CVEs as a function of the time after the last HD session within each group. CVEs consisted of unstable angina, acute myocardial infarction, heart failure exacerbation, transient ischemic attack, stroke, emergent hypertension, and cardiac arrhythmias. CVE diagnoses were obtained from emergency room records and hospital discharge summaries. Data on patient demographics and laboratory values were obtained from emergency room notes, inpatient service notes, and the laboratory section of the electronic medical record.

### Timing of CVEs: observed compared to expected

Time segments of the weekly HD schedule were defined as follows: during and after HD on any HD d as D_0_ (always a ½-d), the d after the last HD as D+1 (either a ½-d or an entire d), the second d after the last HD as D+2 (either a ½-d or an entire d), the third d after the last HD as D+3 (either a ½-d or an entire d), and the fourth d after the last HD as D+4 (always a ½-d before D_0_). Table 1 tabulates how each day of the week consists of D_0_, D+1, D+2, D+3, and D+4 for all 6 weekly HD schedules, and Table 2 demonstrates the percentage of the week consisting of D_0_, D +1, D+2, D+3, and D+4 for all 6 weekly HD schedules. Note that D+4 does not exist for U-IDI (Mon-Wed-Fri and Mon-Tue-Fri). We calculated the observed-to-expected (O/E) CVE ratios during D_0_, D+1, D+2, D+3, and D+4 for U-IDI patients and L-IDI patients as follows. The numerator of the ratio (observed) consists of the fraction of the total number of CVEs that occurred during each time period (ie D_0_, D+1, D+2, D+3, or D+4); and the denominator of the ratio (expected) consists of the fraction of the total number of CVEs that were expected to have occurred at random, ie in proportion to the fraction of the total weeks’ time made up of D_0_, D+1, D+2, D+3, or D+4 (Table 2). The null hypothesis was that the O/E CVE ratio for each weekly time interval (D_0_, D+1, D+2, D+3, D+4) was 1.0.

### Statistical analysis

Normally distributed data were expressed as mean ± standard deviation, and data that were not normally distributed were expressed as median with range. Mortality differences between U-IDI and L-IDI were assessed by a Cox proportional hazards model, in order to account for covariates (coronary artery disease, serum potassium concentration, and serum phosphorus concentration) and Kaplan-Meier estimation (for illustrative purposes). SAS v9.4 was used for statistical analysis. Significance was defined as a p-value of <0.05.

## Results

### Patient demographics and co-morbidities

A total of 149 veterans underwent maintenance outpatient in-center HD at the Ralph H Johnson VAH from January 2002 through October 2015, and 85 met inclusion criteria. All were male. The U-IDI group consisted of 29 veterans with no more than 3 d between HD sessions (Mon-Wed-Fri and Mon-Tue-Fri), and the L-IDI group consisted of 56 veterans with 4 d at times between dialysis sessions (Mon-Wed-Thu, Mon-Tue-Thu, Tue-Wed-Fri, and Tue-Thu-Fri). Approximately 75% of our patients were African-American. Table 3 demonstrates that the demographics and co-morbidities of the 2 groups were similar, except for more coronary artery disease (p=0.02), more glomerular disease as cause of end-stage renal failure (p=0.04), higher serum phosphorus concentration (p=0.005), lower iron saturation (p=0.02), and higher serum potassium concentration (p=0.05) in the U-IDI patients. Laboratory values in Table 3 were averaged over the observation period.

### Mortality in U-IDI and L-IDI

**Figure 1:**
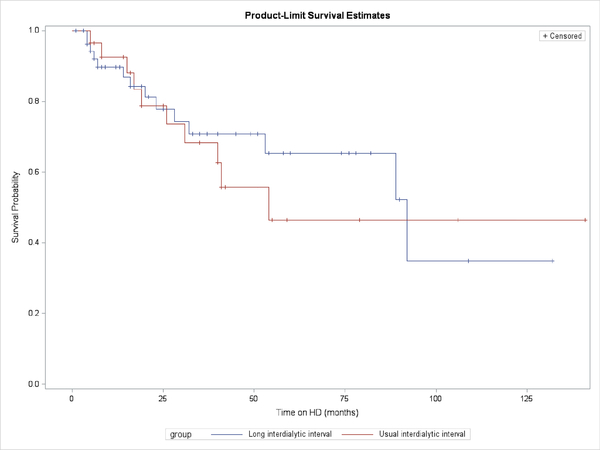
Survival on dialysis: U-IDI patients (blue line) vs L-IDI patients (red line). There is no statistical difference between the lines.

All-cause mortality was not different between the groups, even after normalization for coronary artery disease, serum potassium concentration, and serum phosphorus concentration (Figure 1).

### Occurrence of CVEs

Between 2002 and 2015, 193 CVEs occurred. Some of the 85 patients did not experience a CVE and others experienced multiple CVEs. CVE types consisted of unstable angina or acute myocardial infarction (26%), heart failure exacerbation (29%), transient ischemic attack or stroke (4%), emergent hypertension (28%), and cardiac arrhythmias (%). CVEs occurred more frequency in the U-IDI group than in the L-IDI group (3.7 *vs.* 1.9 events per patient-month, p=0.04).

### Occurrence of CVEs during dialysis and during interdialytic intervals

**Figure 2:**
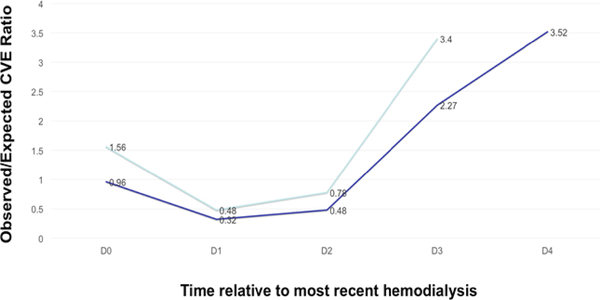
Occurrence of CVEs during HD or at intervals after HD. D0 represents time during HD or the rest of the day after HD. D+1, D+2, D+3, and D+4 represent the first, second, third, and fourth days after HD, respectively. The light blue line represents data from U-IDI patients, and the dark blue line represents data from L-IDI patients. Data are presented as the ratio of the fraction of observed CVEs compared to the fraction of expected CVEs (the latter occurring randomly in proportion to the fraction of the week consisting of each time interval). The ratio of 1 on the y-axis marks CVEs occurring as expected.

We then determined if CVEs were more common as time passed after dialysis; inclusion of L-IDI patients allowed assessment of the longest interdialytic interval, D+4. In separate analyses for U-IDI and L-IDI patients, we calculated the fractions of the total number of CVEs that actually occurred during HD and after HD sessions (D_0_, D+1, D+2, D+3, and D+4) and compared these fractions to the fractions of the total CVEs that were expected to have occurred at random during D_0_, D+1, D+2, D+3, and D+4 (Figure 2). During HD or the remainder of an HD d (D_0_), we observed 56% more CVEs than expected (O/E ratio 1.56) in U-IDI and approximately the same rate of CVEs as expected (O/E ratio 0.96) in L-IDI. Then, we determined if time since the most recent HD, i.e., progressing from D+1 to D+2 to D +3 to D+4, had an effect on CVE occurrence. We observed fewer CVEs than expected on D+1 in both groups: 52% fewer observed than expected (O/E ratio 0.48) in U-IDI and 68% fewer observed than expected (O/E ratio 0.32) in L-IDI. This phenomenon was about the same on the second day after HD (D+2): 22% fewer CVEs observed than expected (O/E ratio 0.78) in U-IDI and 58% fewer CVEs observed than expected (O/E ratio 0.42) in L-IDI. However, as time passed further from dialysis to D+3 and D+4, we observed significantly more CVEs than expected. For D+3, we observed 240% more CVEs than expected (O/E ratio 3.40) in U-IDI and 127% more than expected (O/E ratio 2.27) in L-IDI. For D +4, the longest time interval after the most recent HD, which exit only in the L-IDI group, we observed 252% more CVEs than expected (O/E ratio 3.52). The CVE O/E ratio on D+4 is greater than those during or just after HD (D_0_) and those at lesser times after the most recent HD (D+1, D+2, and D+3).

### Mechanisms of differences in CVE occurrence rates

We then explored mechanisms that might explain the pattern of O/E CVE ratios over the interdialytic time intervals. Mechanisms to consider included elevated serum potassium concentration at the time of the CVE and exaggerated weight (fluid) gain between the last dialysis and the CVE. It was not possible to assess accurately fluid gains (change in body weight from the end of the last HD to the CVE). Weight changes from the end of a HD session to the beginning of the next are accurately and consistently tabulated in the HD unit, but CVEs were defined for this study as events documented in the emergency room or hospital ward. Body weights upon admission to non-HD unit locations were documented intermittently and by scales different from those in the HD unit.

On the other hand, serum potassium concentrations upon presentation to the emergency room or hospital ward for CVEs were readily available, since blood for routine chemistries was drawn from each patient presenting to the emergency room with acute symptoms or exacerbations of chronic diseases. Potassium values on D0 (during or after HD on a HD d) were not relevant, since they would be dropping during HD or would have dropped by the completion of the HD session, respectively. Combining U-IDI and L-IDI patients, we noted a trend towards rising serum potassium concentration (meq/l) at presentation for a CVE with increasing time after HD: 4.7 ± 0.75 (D+1), 5.3 ± 1.3 (D+2), 5.6 ± 1.2 (D+3), and 5.2 ± 0.7 (D+4), with the trend not holding for D+4.

## Discussion

### Major findings

We expected to find greater mortality in patients dialyzing on non-traditional HD schedules with longer interdialytic gaps (L-IDI) than in patients dialyzing on standard HD schedules (U-IDI), but no difference in mortality was observed. We also expected to find a higher incidence of CVEs in L-IDI patients than in U-IDI patients. In contrast, the incidence of CVEs was significantly higher in the U-IDI group, maybe because of more coronary artery disease, hyperphosphatemia, and hyperkalemia in that group. The groups were not matched due to retrospective nature of the study. We next determined, for each group alone, when CVEs occurred as a function of the time relative to HD. CVEs were slightly more likely to occur than expected during and just after HD (D_0_), less likely to occur than expected during the 2 days after HD (D+1 and D+2), and much more likely to occur than expected after longer interdialytic intervals (D+3 and D+4). It is also interesting to note that, in both the U-IDI and L-IDI groups, CVEs were least likely to occur on D+1 and progressively more likely to occur as the time interval from the last HD lengthened, i.e., from D+1 to D+2 to D+3 to D+4.

### Study design

Although our results are in general similar to those from prior studies [1–9], there is novelty in our approach to the question. Our patients were limited to veterans on HD, and the study design was created retrospectively when we realized that an arcane practice in our VAH (no chronic HD on Saturday or Sunday) would allow us to compare outcomes in patients subjected to traditional interdialytic gaps (U-IDI) to outcomes in patients experiencing longer interdialytic gaps (L-IDI). Since patients were not placed on weekly HD schedules for medical reasons and since only HD patients without change in their weekly HD schedules were studied, bias was minimized. In addition, our means of assessing CVE risk for each time period (D_0_, D+1, D+2, D+3, D+4) by comparing the fraction of the total number of CVEs that actually occurred to the fraction of the total number of CVEs that was expected to have occurred (randomly in proportion to the per cent of the week consisting of each time period) is unique.

### Mechanisms

Mechanisms responsible for these results cannot be ascertained from our data. However, it is not surprising that CVEs occurred more frequently than expected during or just after HD (D_0_), when systemic hemodynamics are acutely dysregulated by rapid reductions in serum osmolarity and intravascular volume, when cardiac stunning may occur with overly aggressive ultrafiltration [10], and when cardiac electrical activity might be deranged by acute reductions in serum potassium concentration [15]. Mechanisms responsible for more frequent CVEs long after HD (D+3 and D+4) might be different: arrhythmias from hyperkalemia, pulmonary congestion and hypoxemia after prolonged periods of fluid retention, and myocardial dysfunction caused by increased oxygen consumption in distended cardiac chambers [16]. In addition, the peak concentration hypothesis suggests that a longer interdialytic interval results in accumulation of more uremic toxins, electrolytes and volume that would predispose to CVEs [17].

### Limitations

Conclusions from our data are limited by several factors. The number of patients in our study is small, even though patient data were reviewed since 2002. Patients were placed on HD schedules for non-medical reasons when renal replacement therapy was initiated, but demographics and co-morbidities were not the same in the U-IDI and L-IDI groups. There were higher average serum phosphorus and potassium concentrations, more renal failure caused by glomerular disease, and more coronary artery disease in the U-IDI group. These differences might explain the lack of improved survival and the greater incidence of CVEs in the U-IDI group. The reason for the differences in laboratory values and comorbidities between groups is unclear. It would have been better if accurate weight (fluid) gain data between the end of the last HD and the time of the CVE were available. It should also be noted that the interdialytic intervals for the 2 U-IDI schedules are not the same: Mon-Wed-Fri contains 2 2-d intervals and 1 3-d interval, whereas Mon-Tue-Fri contains 1 1-d interval and 2 3-d intervals. The fact that a number of CVEs were not encountered at our VAH (cardiovascular deaths at home, presentation to other hospitals) renders a degree of inaccuracy to our results and conclusions.

### Implications for clinical practice

Should we extend chronic routine outpatient in-center HD to Saturdays? The answer probably is yes. Out of 75 chronic HD units that are embedded in VAHs, only 5% do not dialyze patients on Saturdays. Beyond the presumed improved medical outcomes resulting from opening our VAH HD unit on Saturdays and thus shortening interdialytic intervals, there are logistic issues to consider. HD nurses might be resistant to extending their workdays from the workweek to Saturdays. On the other hand, dialyzing on an additional day (Saturday) might allow patients whose dialysis has been outsourced to private dialysis companies in the community to return to the VHA HD unit for chronic HD, which could be a money-saving maneuver.

Results of these studies also emphasize the potential value of renal replacement modalities with small interdialytic intervals, such as peritoneal dialysis and home HD [18,19]. Peritoneal dialysis is performed daily, and most home HD patients dialyze 5 times per week, rather than the standard 3 times per week occurring in in-center chronic HD units (Mon-Wed-Fri and Tue-Thu-Sat). It can be seen in Table 2 that traditional in-center HD schedules (U-IDI) result in these weekly percentages for interdialytic intervals: 21.4% (D_0_), 42.9% (D+1), 28.6% (D+2), and 7.1% (D+3). When this calculation is repeated for home HD with 5 sessions per week, for example Mon-Tues-Thu-Fri-Sun, larger weekly percentages for shorter interdialytic intervals are found: 36% (D_0_), 50% (D+1), 14% (D+2), and 0% (D+3). If more CVEs occur as the interdialytic interval lengthens (Figure 2), then peritoneal dialysis and home HD may provide favorable outcomes because of the predominance of shorter interdialytic intervals.

## Figures and Tables

**Table 1:** Daily content of D0, D+1, D+2, D+3, and D+4 for each HD schedule. D (d)=day; D_0_=during and after HD on any HD d; D+1=the d after the last HD; D+2=the second d after the last HD; D+3=the third d after the last HD; D+4=the fourth d after the last HD; U-IDI=usual interdialytic interval; L-IDI=long interdialytic interval; note that each of the 6 HD schedules (rows) totals 7 d.

HD schedule	Mon	Tue	Wed	Thu	Fri	Sat	Sun
Mon-Wed-Fri (U-IDI)	D+3 (½-d) D0 (½-d)	D+1 (entire d)	D+2 (½-d) D0 (½-d)	D+1 (entire d)	D+2 (½-d) DO (½-d)	D+1 (entire d)	D+2 (entire d)
Mon-Tue-Fri (U-IDI)	D+3 (½-d) D0 (½-d)	D+1 (½-d) D0 (½-d)	D+1 (entire d)	D+2 (entire d)	D+3 (½-d) DO (½-d)	D+1 (entire d)	D+2 (entire d)
Mon-Wed-Thu (LIDI)	D+4 (½-d) D0 (½-d)	D+1 (entire d)	D+2 (/-d) D0 (½-d)	D+1 (½-d) DO (½-d)	D+1 (entire d)	D+2 (entire d)	D+3 (entire d)
Mon-Tue-Thu (LIDI)	D+4 (½-d) D0 (½-d)	D+1 (/-d) D0 (½-d)	D+1 (entire d)	D+2 (½-d) DO (½-d)	D+1 (entire d)	D+2 (entire d)	D+3 (entire d)
Tue-Wed-Fri (L-IDI)	D+3 (entire d)	D+4 (/-d) D0 (/d)	D+1 (/-d) D0 (/d)	D+1 (entire d)	D+2 (½-d) DO (½-d)	D+1 (entire d)	D+2 (entire d)
Tue-Thu-Fri (L-IDI)	D+3 (entire d)	D+4 (½-d) D0 (½-d)	D+1 (entire d)	D+2 (½-d) DO (½-d)	D+1 (½-d) DO (½-d)	D+1 (entire d)	D+2 (entire d)

**Table 2:** Each HD schedule’s weekly percentage of D0, D+1, D+2, D+3, and D+4. D (d)=day; D0=during and after HD on any HD d; D+1=the d after the last HD; D+2=the second d after the last HD; D+3=the third d after the last HD; D+4=the fourth d after the last HD; U-IDI=usual interdialytic interval; L-IDI=long interdialytic interval.

HD schedule	D0	D+1	D+2	D+3	D+4	Total
Mon-Wed-Fri (U-IDI)	21.40%	42.90%	28.60%	7.10%	0.00%	100.00%
Mon-Tue-Fri (U-IDI)	21.40%	35.70%	28.60%	14.30%	0.00%	100.00%
Mon-Wed-Thu (L-IDI)	21.40%	35.70%	21.40%	14.30%	7.10%	100.00%
Mon-Tue-Thu (L-IDI)	21.40%	35.70%	21.40%	14.30%	7.10%	100.00%
Tue-Wed-Fri (L-IDI)	21.40%	35.70%	21.40%	14.30%	7.10%	100.00%
Tue-Thu-Fri (L-IDI)	21.40%	35.70%	21.40%	14.30%	7.10%	100.00%

**Table 3:** Patient characteristic (at initiation of HD) and average laboratory values (over the entire HD treatment period) for U-IDI and L-IDI groups.

Parameter	U-IDI group (n=29)	L-IDI group (n=56)	p-value
**Time on HD (months)**	26 (5–141)	20 (1–132)	0.97
**Age at start of HD (years)**	59 (38–82)	62 (40–85)	0.71
**Ethnicity [# of patients (%)]**			0.06
White	10 (34%)	10 (18%)	
African-American	18 (62%)	46 (82%)	
Other	1 (4%)	0 (0%)	

**Co-morbidities [# of patients (%)]**			
Smoking	11 (38%)	15 (27%)	0.29
Diabetes mellitus	19 (66%)	40 (71%)	0.58
Hypertension	27 (93%)	55 (98%)	0.27
Peripheral vascular disease	7 (24%)	12 (21%)	0.78
Coronary artery disease	15 (52%)	15 (27%)	0.02
Heart failure	12 (41%)	22 (39%)	0.85
Cerebrovascular accident	4 (14%)	9 (16%)	1
Arrhythmias	7 (24%)	7 (13%)	0.17
Glomerular disease	5 (17%)	2 (4%)	0.04
Cancer	6 (21%)	11 (20%)	0.91
Hyperlipidemia	16 (55%)	35 (63%)	0.51

**Laboratory values**			
Kt/V (single pool)	1.6 ± 0.1	1.5 ± 0.2	0.11
Hemoglobin (g/dl)	10.9 ± 1.0	10.6 ± 1.1	0.23
Ferritin (mcg/l)	575 ± 238	520 ± 439	0.46
Iron saturation (%)	32 ± 9	38 ± 15	0.02
Calcium (mg/dl)	8.7 ± 0.5	8.8 ± 2.9	0.19
Phosphorus (mg/dl)	5.8 ± 1.4	4.8 ± 0.9	0.005
Parathyroid hormone (pg/ml)	488 ± 397	490 ± 376	0.98
Albumin (g/dl)	3.3 ± 1.2	3.3 ± 0.5	0.71
Potassium (meq/l)	4.6 ± 0.5	4.4 ± 0.5	0.05

**Deaths (# of patients)**	10	14	0.45
